# Assessing inhabitants’ satisfaction and service needs: a cross-sectional survey of primary care service in Jiangsu Province, China

**DOI:** 10.1017/S1463423625100558

**Published:** 2025-11-07

**Authors:** Wenjun Yan, Vorapoj Promasatayaprot, Sumattana Glangkarn

**Affiliations:** 1 School of Public Health, Xuzhou Medical University, Jiangsu, China; 2 Faculty of Public Health, Mahasarakham University, Mahasarakham, Thailand

**Keywords:** cross-sectional survey, healthcare needs, Jiangsu Province, patient satisfaction, primary care

## Abstract

**Background:**

Primary care serves as the foundation of a well-functioning healthcare system and is critical for ensuring equitable access, early disease management, and cost-effective service delivery. In China, reforming primary-level healthcare institutions has become a national priority to achieve universal health coverage. Understanding the service users’ perspectives is essential to align primary care services with population health needs and improving trust in grassroots healthcare providers.

**Aim:**

To evaluate satisfaction and unmet service needs among primary care users in Jiangsu Province, China.

**Methods:**

A cross-sectional survey using structured questionnaires was given to 424 residents in urban and rural Jiangsu Province to measure satisfaction with primary hospitals, doctors, service preferences, and perceived gaps. Data was analyzed using descriptive statistics and chi-square tests to identify regional differences.

**Results:**

The primary care utilisation rate among respondents was 73.3%. While 75.2% reported satisfaction with medical expenses at primary-level facilities, significant urban-rural and regional differences were observed in service awareness, health policy knowledge, and perceived quality of doctor-patient communication. Primary care doctors received the highest ratings for using “easy-to-understand language”(mean score 4.13 ± 0.821) but lower scores for “professional competence” and “treatment effectiveness” (both 3.91). Rural inhabitants expressed high demand for services like management of common diseases (65.8%) and routine health examinations (52.4%), but highlighted shortages in advanced diagnostic tools (e.g., CT scans, endoscopy). Most inhabitants (67.2%) felt that primary care providers require improvement, particularly in clinical competency and communication.

**Conclusion:**

The findings highlight areas for targeted policy interventions to improve primary care service delivery and capacity-building of primary care doctors in Jiangsu Province.

## Introduction

Primary care remains a global priority, including in China (Central Committee of the Communist Party of China and State Council, 2016), Its quality directly impacts health outcomes (World Health Organization, 2018). As a people-centered service, primary care must prioritize user perceptions.

Patient satisfaction serves as a key quality metric (Umoke *et al*., 2020), with global studies focusing on this theme (Lian and Wilsgaard, 2004; Dong *et al*., 2017; Ferreira *et al*., 2020). More research has focused on identifying the influencing factors of satisfaction—variables associated with inhabitant s’ satisfaction with primary care through mechanisms like demand-supply alignment, service experience, or interaction quality. Demographic characteristics include several key influencing factors: Education level has been found to negatively correlates with inhabitants’ satisfaction with primary care, as shown in multiple surveys (Li, 2021; Zhao *et al*., 2021; Liu *et al*., 2022; Ren *et al*., 2022; Wang, 2023)—a pattern attributed to higher education levels raising expectations for medical services, creating a gap between demand and perceived supply. A similar association exists for monthly income (Wang, 2023; Zhang *et al*., 2021), with higher income often linked to greater service expectations. Other demographic variables associated with satisfaction include gender (Xiao, 2021; Ren *et al*., 2022), age (Xiao, 2021; Wang, 2023), and health status (Xiao, 2021; Liu *et al*., 2022). Specifically, elderly inhabitant s, female inhabitant s, and those with poor health conditions tend to report higher satisfaction, potentially due to closer alignment between their needs (e.g., frequent care access, emotional support) and primary care services. Healthcare accessibility is another critical influencing factor: Countries where inhabitants access medical resources without referral report higher satisfaction than those requiring referrals (Kroneman *et al*., 2006), reflecting the role of service availability in shaping experience. Insurance type also matters—for instance, in the US, individuals with employer-sponsored insurance are less satisfied than those with Medicare (Wray *et al*., 2021), likely due to differences in coverage scope and service constraints. Regarding service quality itself, several interactional and experiential factors influence satisfaction: The adoption of “person-centered” care models (Detollenaere *et al*., 2018), the development of empathy in doctor-patient interactions (Watts et al., 2023), and patients’ perceived quality of received services (Wang, 2023; Zhang *et al*., 2023) all correlate with higher satisfaction. Notably, trust—often fostered by perceived quality and empathetic communication (Xiao, 2021; Wang, 2023)—emerges as both an outcome of positive interactions and an influencing factor in its own right, reinforcing satisfaction through enhanced treatment adherence and perceived reliability.

From a global perspective, satisfaction varies greatly: a study spanning 31 European countries shows high satisfaction rates in primary healthcare in Europe: 92.1% of the respondents are satisfy with the primary care they receive (Detollenaere *et al*., 2018), while a study form Ukraine shows that: inhabitants satisfaction rate is 75.3% (users) and 71.9% (nonusers)(Anufriyeva *et al*., 2023), similarly, a meta-analysis of 26 cross-sectional studies involving 36,430 participants (Zhang *et al*., 2021) reported a satisfaction rate of 77.3% in China. Even in China, satisfaction also varies a lot: a study in Shanghai showed high scores of satisfaction (97.01) (Han *et al*., 2022), but in a Shandong research, only 66.91% inhabitants are satisfied with the local primary care (Ren *et al*., 2022).

The most significant dimension explaining overall satisfaction index is the satisfaction regarding care quality (Ferreira *et al*., 2020). A survey based on cross-sectional data of 2016 China Family Panel Studies shows that in rural areas of China, inhabitants are more satisfied with infrastructure than with service quality in primary care (Xiao, 2021). Another local survey in Hubei Province (Ao *et al*., 2018) shows that the highest satisfaction among inhabitants lies in the convenience and privacy protection of medical treatment, while the lowest satisfaction lies in service quality and facilities in primary care.

In recent years, the Chinese government has done a lot of work to improve the service capacity of primary care, such as introduced a hierarchical medical system (General Office of the State Council, 2015), increasing the training of grassroots doctors (The Development and Reform Commission of the Ministry of Education, 2015) and so on. As one of the most economically developed provinces in China， Jiangsu Province also focused on improving the quality of primary health care (Health Commission of Jiangsu Province, 2019) from 2019-2023, the new policy aimed to improve the salary of grassroots doctors, strengthen their on-the-job training and expand their development space. After all the efforts made by the national and local governments, how satisfied are the local people with primary healthcare? It is necessary to do some research on this matter.

Most previous studies have investigated inhabitants’ views on the existing medical system (Li *et al*., 2021; Kalkman *et al*., 2022; Zhang *et al*., 2023), but we should know what are the inhabitants’ needs (Starfield, 2009), what their ideal primary care looks like. This study aims to investigate the satisfaction of inhabitants in Jiangsu Province for primary care and outline the needs of inhabitants for primary care.

## Methods

### Study design

This cross-sectional study was conducted to assess residents’ satisfaction with and needs for primary care across Jiangsu Province from October to November 2023. The design was chosen to capture population-level attitudes at a single time point following recent healthcare reforms, to enable urban–rural comparisons, and to identify immediate service gaps for policy action.

### Setting

Jiangsu is a highly developed province, but its economic growth is uneven. Northern Jiangsu (per capita GDP: 75,551 RMB) comprises Xuzhou, Lianyungang, Yancheng, Huai’an, and Suqian; Central Jiangsu (per capita GDP: 123,551 RMB) includes Yangzhou, Nantong, and Taizhou; and Southern Jiangsu (per capita GDP: 167,995 RMB) encompasses Suzhou, Wuxi, Changzhou, Zhenjiang, and Nanjing (Yan and Sun, 2022). Like the rest of China, Jiangsu lacks a gatekeeping system for primary care; inhabitant s are not required to obtain a referral from a general practitioner before accessing secondary or tertiary hospitals (Yan *et al*., 2025). Consequently, a variety of factors influence the rate at which inhabitant s choose to seek care at the primary level.

### Participants and recruitment

The study population included urban and rural inhabitants in Jiangsu Province.

Inclusion criteria:

1. Over 16 years old;
2. The administrative division of residence is in Jiangsu Province;
3. Able to read and understand Chinese mandarin independently or with the help of others;
4. Willing to participate.

Exclusive criteria:

1. Under the age of 16;
2. Refuse to participate in the investigation;
3. Unable to participate in the survey completely.

Sampling size:

The sample size was estimated using the following formula

Sample size = 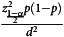 , in which (Charan and Biswas, 2013)

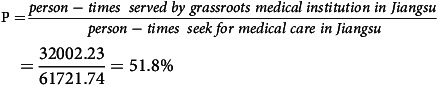




Sample size = 
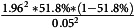
 =383 (Jiangsu Provincial Health Commission, 2020)

The calculated sample size was 383 cases. To account for potential data loss during collection, the sample size was increased by 15%, resulting in a final sample of 441 participants.

Sampling methods:

A mixed sampling approach combining cluster sampling and convenience sampling was employed to ensure both diversity and representativeness. Initially, cluster sampling was utilized to randomly select 7 out of 15 clinical medicine classes from Xuzhou Medical University, yielding 147 student participants. These students, all native to Jiangsu Province, demonstrated high demographic homogeneity with provincial inhabitants, thus serving as representative samples of Jiangsu’s diverse regional populations. On this basis, convenience sampling was integrated to expand the survey scope. Students were instructed to distribute three questionnaires to their neighbors during holiday visits and return completed surveys to the research team upon reenrollment. To enhance representativeness, specific inclusion criteria were enforced, requiring students to target respondents across gender, age groups, and socioeconomic strata. This combined methodology effectively captured multi-dimensional demographic characteristics, minimized sampling bias, and mitigated selection bias arising from subjective participant selection.

### Research instrument

The questionnaire was developed by a panel of three experts: a primary-care administrator, a general practitioner, and a health-policy researcher. It consists of four sections:

Section 1. Demographic characteristics: place of residence, household registration status, gender, age, education level, monthly income, medical insurance, chronic-disease status, and self-rated health.

Section 2. Perceptions of primary-care services: choices for minor-illness care, knowledge of China’s hierarchical medical system, attitudes toward the “first-visit at primary-care” policy, views on the doctor–patient relationship in primary care, perceived role of primary-care doctors, opinions on the price and variety of medicines, adequacy of medical equipment, and evaluations of the consultation process and physical environment.

Section 3. Satisfaction with primary-care doctors. This section contains 21 items grouped into three dimensions, detail can be check in Table 1. rated on a 5-point Likert scale: 5 = excellent, 4 = good, 3 = average, 2 = poor, 1 = very poor. Cronbach’s α for the scale was 0.991, indicating excellent internal consistency.

**Table 1.** Dimensions and items of satisfaction with primary-care doctors

Dimension	Item
Attitude	Have a kind attitude during therapy
	Easygoing language during consultation
	Focus while treating
	Patience to communicate with
	Mutual respect
	Protect your privacy
	Adequate visit time
Service capability	Detailed consultation during treatment
	Professional
	Clear and understandable disease explanation
	Definite diagnosis
	The diagnosis and treatment process is crisp and smooth
	Effective treatment
Need meet	Timely reception
	Answer questions in time
	Meet your prescription requirements
	Meet your inspection requirements
	Meet your health education needs
	Choose the right treatment for you based on the financial situation
	The treatment plan respects the choice of patients and their families

Section 4. Inhabitants’ needs for primary-care services, assessed through four key questions:

1. Which examinations have you received at primary-care facilities and which additional examinations would you like to be offered?
2. Which services do you expect primary-care facilities to provide?
3. What aspects of primary-care services need improvement?
4. What do you consider to be the responsibilities of primary-care doctors?

For these four questions, the same three experts rated each item on relevance, clarity, and completeness. The item-level content validity index (I-CVI) for every item exceeded 0.83, demonstrating good content validity.

### Data collection

The 147 introducers were trained before national holiday. Then they went home and distributed the questionnaire to one of their relatives and submit the questionnaire before the end of national holiday. Data collection took place from October to November 2023. 441questionaires were collected, excluding 17 from outside Jiangsu Province, a total of 424 valid questionnaires were obtained, the response rate is 96.1%.

### Data analysis

IBM’s SPSS version 23.0 will be used to analyze all quantitative data. Descriptive statistics were performed for describe demographic characteristics variables. Chi-square tests were used to compare urban–rural differences in perceptions of primary-care services across the three regions. Means and standard deviations were used to describe satisfaction for grassroots doctors. The statistically significant level was set as *P* < 0.05.

### Ethical considerations

The study has approved the review of Ethics Committee of Mahasarakham University (Reference Number 409–363/2023) and Xuzhou Medical University (Reference Number XZHMU-2023088). All participants gave verbal informed consent after the aims, procedures, and their right to withdraw at any time had been explained. No personal identifiers were recorded; all data were anonymised and stored on a password-protected server accessible only to the research team.

## Results

### Demographic characteristics

Among the 424 respondents, 217 (51.2%) were from northern Jiangsu Province, 50 (11.8%) from Central Jiangsu Province, 157 (37%) from southern Jiangsu Province, 243 (57.3%) from urban areas, 181 (42.7%) from rural areas, 266 (62.7%) female and 158 (37.3%) male. The age range was from 16 to 86 years (25th percentile = 21 years, 50th percentile = 23 years, 75th percentile = 45 years), 12(2.8%) had a primary school or lower education level, 37(8.7%) had a junior high school education level, 80(18.9%) had a high school or vocational school education level, 51(12%) had graduated from vocational colleges and 244 (57.4%) had a bachelor’s degree or above.

Respondents’ monthly income distribution ranged from 0 yuan to 125,400 yuan (25th percentile = 0 yuan, 50th percentile = 1850 yuan, 75th percentile = 5000 yuan), and average monthly personal medical expenses ranged from 0 yuan to 10,000 yuan (25th percentile = 0 yuan, 50th percentile = 50 yuan, 75th percentile = 200 yuan). 233 respondents (53.6%) were covered by employee medical insurance, 155 (3.6%) were covered by inhabitant medical insurance, 40 (9.4%) were covered by rural cooperative medical insurance, and 6 (1.4%) were beneficiaries of subsistence allowances.

Of the 424 inhabitants surveyed, 76 (17.9%) had chronic diseases, of which 37 (8.7%) had high blood pressure and 12 (2.8%) had diabetes. In the past three months, 155 people (36.6%) claimed to be in very good health, 165 (38.9%) claimed to be in good health, 96 (22.6%) claimed to be in average health, and 11(2.6%) claimed to be in poor health.

### Perceptions of primary-care services

Among the surveyed inhabitants, 311 (73.3%) had been treated in primary-level hospitals, of which 64.2% were treated in urban areas and 85.6% in rural areas, the walk time from home to the nearby primary-level hospitals ranged from 0 to 180 minutes(25th percentile = 7 minutes, 50th percentile = 10 minutes, 75th percentile = 20 minutes). When encountering small health issues such as cold and fever, 223 respondents (52.5%) would choose the primary-level hospital near their home. 239 (56.3 %) were familiar or know what the hierarchical medical system is, and 325 (76.7%) supported it. 286 people (67.5 %) believed that primary-level hospitals could play a certain role, but the role was limited. Details can be check in Table 2.

**Table 2.** Views of urban and rural inhabitants in different regions on primary-level hospitals

	Northern Jiangsu				Middle Jiangsu				South Jiangsu				*χ*^2^	*P*
	Urban	Rural	*χ*²	*P*	Urban	Rural	*χ*²	*P*	Urban	Rural	χ²	P		
**With small health issues**														
Primary-level hospital	54	64	11.499	0.001	8	19	3.678	0.055	43	35	4.919	0.027	20.107	< 0.001
Tertiary hospital	32	7	12.888	< 0.001	3	5	0.080	0.777	26	11	0.905	0.341		
Pharmacy	84	53	3.916	0.048	12	19	0.363	0.547	66	35	0.334	0.563	10.033	0.002
**Hierarchical medical system in China**														
Familiar with	16	14	12.494	0.006	3	1	6.199	0.102	12	5	2.926	0.403	14.142	0.003
Know	61	37			11	14			42	23				
Heard of	41	28			7	10			36	18				
Not know	4	16			0	4			10	11				
**The policy “first visit in primary-level hospital”**														
Support	86	76	2.584	0.275	20	25	2.384	0.304	78	40	1.196	0.550	0.299	0.861
Unsupported	18	10			1	2			14	11				
Not know	18	9			0	2			8	6				
**Whether you have been to a primary hospital**														
Yes	67	82	24.471	< 0.001	13	26	3.968	0.046	76	47	0.892	0.345	24.388	< 0.001
No	55	13			8	3			24	10				
**Views on Primary-level hospitals**														
Very useful	33	31	0.909	0.635	5	6	1.143	0.565	39	19	0.500	0.479	0.631	0.729
Limited role	87	62			16	22			61	38				
Useless	2	2			0	1			0	0				
**Relationship with patients**														
Very harmonious	26	31	12.217	0.002	4	6	1.146	0.564	26	14	2.196	0.533	9.168	0.027
Harmonious	94	55			17	22			70	39				
Tense	2	9			0	1			4	3				
Very tense	0	0			0	0			0	1				
**Maintaining inhabitants’ health**														
Play a great role	28	29	4.283	0.117	8	3	6.787	0.079	28	16	4.138	0.247	6.252	0.100
Play a part	82	51			11	23			62	30				
Very limited role	12	15			2	2			10	10				
Mostly useless	0	0			0	1			0	1				
**Quality of grassroots doctors**														
Education level	33	12	10.480	0.015	3	1	7.805	0.050	15	10	0.483	0.923	12.890	0.005
Experience	72	58			16	17			68	36				
Attitude	14	18			2	8			16	10				
Other	3	7			0	3			1	1				

**Figure 1.**
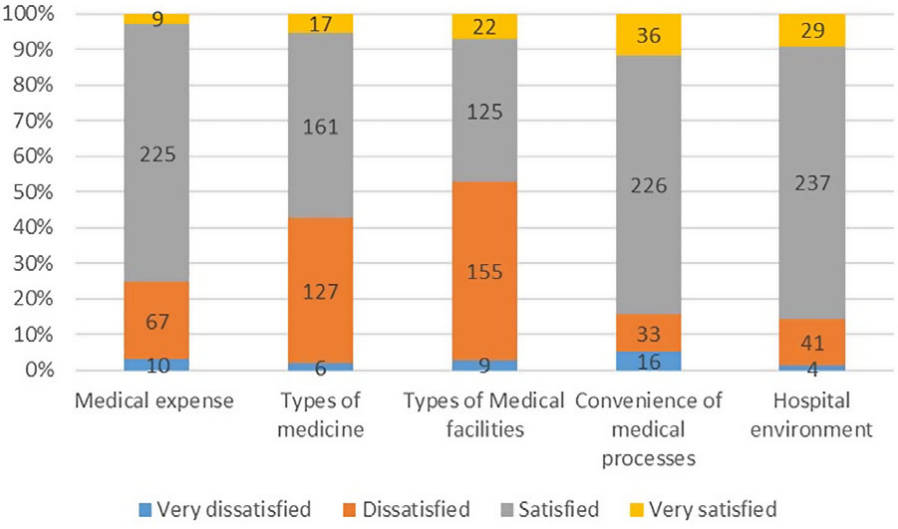
Satisfaction of inhabitants on price of medicine, types of medicine, types of medical facilities, convenience of medical processes and hospital environment. Stacked-bar chart showing the distribution of satisfaction levels among Jiangsu residents regarding five aspects of primary care. Bars represent the percentage of respondents who were “very dissatisfied” (blue), “dissatisfied” (orange), “satisfied” (gray), or “very satisfied” (yellow) for each dimension.

The waiting time of the respondents ranged from 0 minutes to 180 minutes (25th percentile = 5 minutes, 50th percentile = 10 minutes, 75th percentile = 20 minutes). 234(75.2%) respondents claimed satisfied or very satisfied with the medical expense in Primary-level hospitals. The satisfaction rate (satisfied and very satisfied) is 178(57.3%), 147(47.3%), 262(84.3%) and 266(85.5%) on price of medicine, types of medicine, types of medical facilities, convenience of medical processes and hospital environment separately. Detail can be check in Figure 1.

### Satisfaction with primary-care doctors

We assume that the main problem of primary care is the quality of services provided by grassroots doctors, then, a survey was conducted on inhabitants’ satisfaction with grassroots doctors. The satisfaction scale was divided into three dimensions: attitude, service capability and need meet. The score was calculated by “very good” (5 points), “good” (4 points), “average” (3 points), “poor” (2 points) and “very poor” (1 point). This study also conducted statistical tests on different regions and between urban and rural areas, and the results showed no significant statistical difference, so only statistical description was given here. The results showed that the scores of agreeableness were the highest in consultation (4.13 ± 0.821), and the scores of disease explanation (3.91 ± 0.884) and treatment effectiveness (3.91 ± 0.845) were the lowest, as shown in Table 3.

**Table 3.** Inhabitants’ satisfaction with primary care doctors

		Scores
Attitude	Have a kind attitude during therapy	4.10 ± 0.821
	Easygoing language during consultation	4.13 ± 0.821
	Focus while treating	4.08 ± 0.807
	Patience to communicate with	4.04 ± 0.843
	Mutual respect	4.09 ± 0.839
	Protect your privacy	4.10 ± 0.803
	Adequate visit time	4.03 ± 0.847
Service capability	Detailed consultation during treatment	3.98 ± 0.860
	Professional	3.91 ± 0.884
	Clear and understandable disease explanation	3.98 ± 0.852
	Definite diagnosis	3.94 ± 0.861
	The diagnosis and treatment process is crisp and smooth	4.01 ± 0.824
	Effective treatment	3.91 ± 0.845
Need meet	Timely reception	4.06 ± 0.791
	Answer questions in time	4.04 ± 0.827
	Meet your prescription requirements	4.01 ± 0.828
	Meet your inspection requirements	3.96 ± 0.840
	Meet your health education needs	3.96 ± 0.847
	Choose the right treatment for you based on the financial situation	3.98 ± 0.829
	The treatment plan respects the choice of patients and their families	4.06 ± 0.810

### Inhabitants’ needs for primary-care services

**Figure 2.**
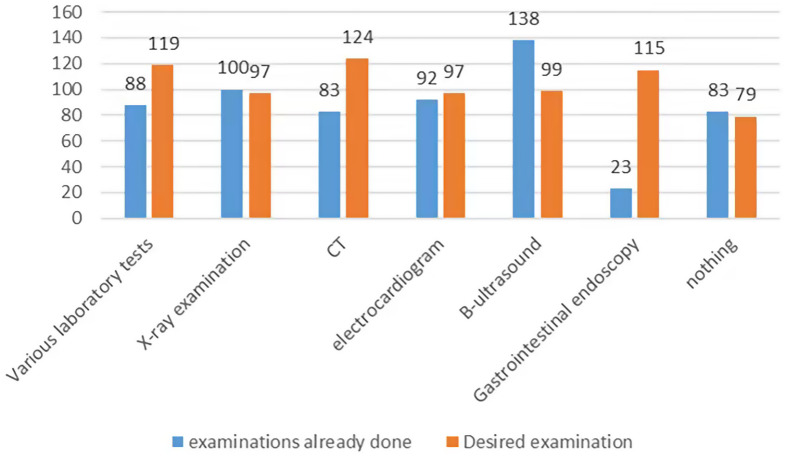
Demand for various examinations in primary-level hospitals (number of people). Demand for selected diagnostic examinations in primary-level hospitals among surveyed residents of Jiangsu Province. Bars indicate the absolute number of respondents who reported having already undergone (blue) or still desired (orange) each examination type.

At the end of the study are four questions what we highlight in this study. When asked what examinations they have undergone in primary-level hospitals and what examinations they hope to do, the most common examinations are B-ultrasound, X-ray examination and electrocardiogram, while the demands of CT, various laboratory tests and gastrointestinal endoscopy exceeded supply. The responses of respondents are shown in Figure 2.

**Figure 3.**
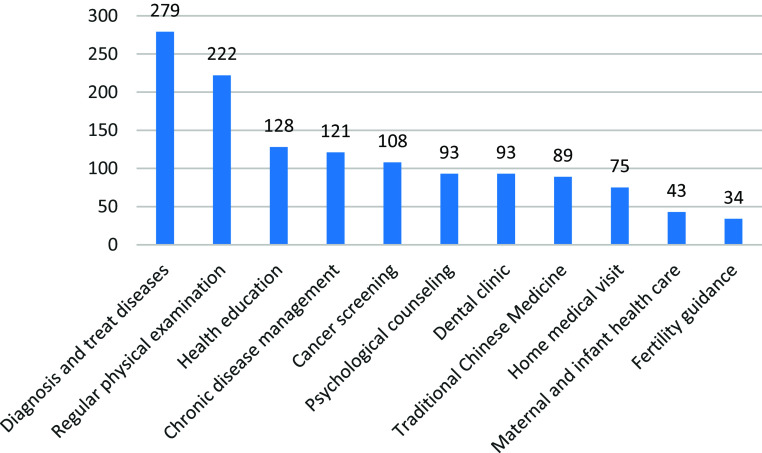
Services need to be provided in primary-level hospitals (number of people). Services that respondents most wanted primary-level hospitals to provide. Bars show the absolute number of residents who expressed a need for each service.

“Diagnosis and treat disease” (279(65.8%)) and “Regular physical examination” (222(52.4%)) were top two ranked answers of the question “What services do you hope primary-level hospitals provide for you?” Detail is in Figure 3.

**Figure 4.**
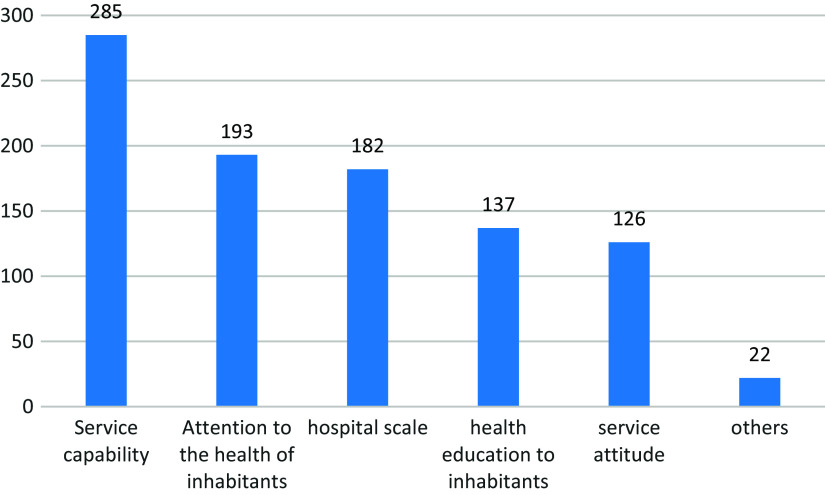
Areas for improvement in primary-level hospitals (number of people). Areas identified by residents as needing improvement in primary-level hospitals. Bars represent the absolute number of respondents who selected each domain.

When asked about the aspects that need to be improved in primary-level hospitals, 285 (67.2%) inhabitants said that the service capability of grassroots doctors needs to be improved, and the specific views of the surveyed inhabitants are shown in Figure 4.

**Figure 5.**
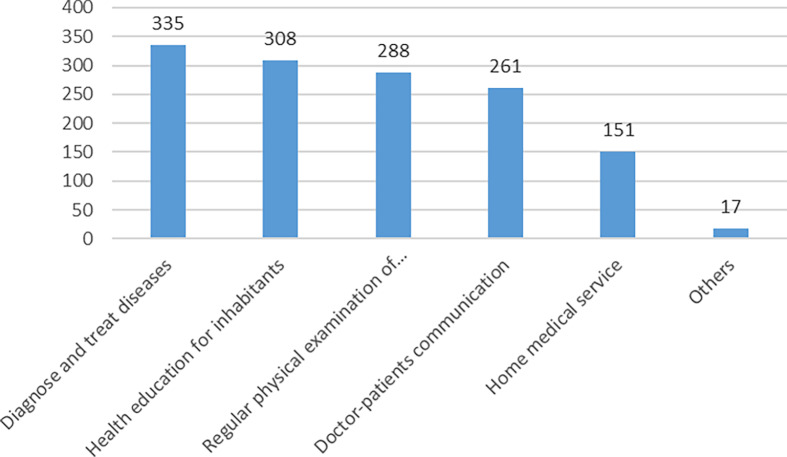
The responsibilities of primary care doctors according to the respondents. Responsibilities that respondents expect primary care doctors to fulfill. Bars indicate the absolute number of respondents selecting each duty.

For the responsibilities of grassroots doctors, the top priority among inhabitants is still to diagnose and treat diseases (335 (79.0%)), followed by the health education to inhabitants (308(72.6%)) regular physical examination of inhabitants (288(67.9%)) and doctor-patient communication (261(61.6%)), as shown in Figure 5.

## Discussion

This study aimed to evaluate the satisfaction and needs of inhabitants in Jiangsu Province regarding primary healthcare services, against the backdrop of ongoing national and local efforts to strengthen the primary care system.

## Primary visiting rate and regional disparities

The primary care visit rate in Jiangsu Province (73.3%) is higher than the national average (61.4%) reported in a meta-analysis of 26 cross-sectional studies (Zhang *et al*., 2021), which may be related to Jiangsu’s economic development and local policy support for primary healthcare (Health Commission of Jiangsu Province, 2019). Notably, the rural visit rate (85.6%) is significantly higher than the urban rate (64.2%), consistent with a previous survey in Xuzhou, Jiangsu (Miao *et al*., 2019), where the rural primary care visit rate reached 82.1%. This phenomenon does not imply superior rural primary care quality but rather reflects limited medical options for rural inhabitant s geographical and transportation constraints force them to rely on nearby primary care institutions. In contrast, urban inhabitant s, especially those in northern Jiangsu, prefer tertiary hospitals for minor illnesses, likely due to greater accessibility to high-level medical resources (Yan *et al*., 2025).

The awareness rate of the hierarchical medical system in Jiangsu (44.3%) is similar to that in Weifang, Shandong (40.4%) (Liu *et al*., 2022), with rural inhabitant s, particularly in northern Jiangsu, showing lower awareness. This indicates insufficient publicity of the hierarchical medical system, as noted in previous studies (Zhao *et al*., 2021), where low policy awareness hinders inhabitant s’ adherence to first visit at primary care and weakens the effectiveness of graded diagnosis and treatment.

## Satisfaction with primary care services and doctors

Inhabitant s’ high satisfaction with medical expenses in primary care institutions (75.2%) aligns with the emphasis on affordable healthcare in China’s primary care policies (General Office of the State Council, 2015). However, satisfaction with drug variety (47.3%) and medical equipment (57.3%) is relatively low, consistent with findings in western Guangxi (Zhang *et al*., 2022), where unreasonable allocation and low utilization of primary medical facilities led to low inhabitant satisfaction (26%).

This study highlights what kind of grassroots doctors the inhabitants really need. 67.2% of the inhabitants thought that the quality of grassroots doctors needed to be improved urgently. This indicates that grassroots doctors are not trusted by the inhabitants. In the opinions of 80% of the inhabitants, the primary responsibility of grassroots doctors is disease diagnosis and treatment, followed by health education to inhabitants regular physical examination and doctor-patient communication. The competence of doctors is the “primary productivity” of primary care: a Portuguese study (Ferreira *et al*., 2020) showed that the main contribution to overall patient satisfaction came from satisfaction with the services provided by general practitioners. In her research on the influencing factors of satisfaction with basic medical and health services, a Chinese author (Yang, 2023) also pointed out that the effect of medical services and the satisfaction with medical and health services are the most significant. A study covering 31 countries in Europe (Detollenaere *et al*., 2018) pointed out: On average, 93.2% of the European respondents were satisfied with their GP. A study on hospital satisfaction in rural areas in Nigeria (Umoke *et al*., 2020) showed that patients’ satisfaction with physician reliability ranked behind dimensions such as responsiveness and empathy, although they claimed to be satisfied with reliability as well. In China, a study (Ren *et al*., 2022) in Shandong Province shows that inhabitants are highly satisfied with the diagnosis and treatment effect and service attitude of primary care. However, in the satisfaction survey involved in this study, the inhabitants are most satisfied with “the service attitude of primary doctors”, while the satisfaction with “professional disease explanation and effective treatment” is low. It can be seen from this that the service satisfaction of grassroots doctors varies greatly not only in the world, but also in China. On the one hand, this is due to the different evaluation indicators used in different studies, and on the other hand, it shows that the competency of grassroots doctors trained by different systems is also different.

## Inhabitant s’ needs and implications for service improvement

Inhabitant s’ top demands for primary care include “diagnosis and treatment of common diseases”(65.8%) and “regular physical examinations”(52.4%), consistent with the core role of primary care in basic health services (World Health Organization, 2018). Additionally, 67.2% of inhabitant s believe grassroots doctors’ service capabilities need improvement, echoing (Yang, 2023)’s conclusion that medical service effectiveness is the most significant predictor of satisfaction.

Inhabitant s also emphasize that grassroots doctors should prioritize “disease diagnosis and treatment,” “health education,” “regular physical examinations,” and “doctor-patient communication.” This aligns with the “people-centered” primary care model (Detollenaere *et al*., 2018), indicating that primary care in Jiangsu should shift from a focus on treatment alone to integrated health management.

Notably, demand for CT, laboratory tests, and gastrointestinal endoscopy in primary care exceeds supply. This gap may stem from two factors: first, inhabitant s’ lack of clarity on the necessity of certain examinations, highlighting the need for strengthened health education (Starfield, 2009); second, inadequate integration of high-level medical resources into primary care, contradicting the goal of the hierarchical medical system to share examination resources between primary and tertiary hospitals (General Office of the State Council, 2015).

## Policy and practical implications

The findings of this study highlight actionable areas where targeted interventions could strengthen primary healthcare in Jiangsu Province.

1. Enhancing Medical Education and Training: Strengthening the clinical skills of grassroots doctors through targeted training programs and continuing education is essential to improve trust and treatment outcomes.
2. Improving Resource Allocation: Expanding access to diagnostic tools and integrating primary care with higher-level hospitals could bridge the gap between demand and supply for advanced services.
3. Public Education Campaigns: Increasing awareness of the hierarchical medical system and the role of primary care could shift patient behavior toward more appropriate utilization of healthcare resources.

## Limitations

The study’s generalizability is constrained by its relatively small sample size (*N* = 424) and geographic concentration in Jiangsu Province, which limits applicability to broader Chinese populations. Data collection relied on self-reported questionnaire surveys, introducing potential recall bias and subjective response distortion that may compromise measurement accuracy. Convenience sampling additionally risked over-representing younger, more educated demographic subgroups with greater likelihood of interacting with student recruiters. Moreover, the cross-sectional design precluded temporal analysis, preventing examination of longitudinal trends in primary healthcare utilization patterns.

## Conclusion

The high primary visiting rate in rural Jiangsu underscores the importance of ensuring quality care in these areas, while urban disparities highlight the need to redirect patient flows to primary facilities. Addressing the competency of grassroots doctors, expanding diagnostic capabilities, and improving policy awareness are critical steps toward building a robust primary care system that meets the needs of all inhabitants. Future efforts should prioritize these areas to achieve the goals of the hierarchical medical system and enhance overall healthcare satisfaction.

## Data Availability

The datasets generated and analyzed during the current study are not publicly available but are available from the corresponding author on reasonable request.
